# Scrotal leiomyoma: A rare benign scrotal wall lesion – A case report^☆^

**DOI:** 10.1016/j.eucr.2026.103386

**Published:** 2026-02-21

**Authors:** Vikram Lal Seetlani, Muhammad Waqas Shafiq, Syed Adeel Ahmed, Muhammad Bilal Rasheed, Faisal Rehman, Muhibullah Bangash, Khurram Mir, Mohammad Usman Khan

**Affiliations:** a Urology Department, Pakistan; b General Surgery Department, Pakistan; c Pathology Department, Pakistan

## Abstract

Leiomyomas are uncommon benign tumors of the genitourinary system that can mimic benign scrotal lesions such as sebaceous cysts. A 56-year-old male with a 20-year history of a painless scrotal swelling. It was hard, irregular, non-tender swelling in lower midline of scrotum, distinct from both testes and spermatic cords. He underwent complete surgical excision of a 4 x 5cm lesion. Histopathology confirmed a benign leiomyoma without atypia or any mitotic activity. There was no recurrence at 1 year follow-up. Scrotal leiomyomas is one of the differentials in a long-standing scrotal swelling. Surgical excision is both diagnostic and curative.

## Highlights

• Leiomyoma can be one of the differential of chronic scrotal lesions in middle aged men.
• The histopathologist must carefully assess for the subtle signs of malignancy.
• Start withonservative management and surgical excision can be considered if enlarging and symptomatic.

## 1 Introduction

Scrotal leiomyomas are extremely rare, comprising less than 1% of genitourinary tumors.^1^ These benign lesions of smooth muscle origin, are commonly found in uterus, gastrointestinal tract, and skin 2. They are slow-growing, painless masses, mimicking other benign scrotal lesions such as sebaceous cysts, lipomas, epididymal lesions.^3^ Due to there rarity, preoperative diagnosis is challenging, and histopathology remains the gold standard for confirmation.^4^ We present a case of scrotal leiomyoma with a 20-year history of an indolent scrotal swelling.

## 2 Case presentation

A 56-year-old male presented with a slowly enlarging, painless swelling in the lower midline of scrotum for 20 years. There was no history of trauma, fever, urinary complaints, or systemic symptoms. He is a known smoker. On physical examination a firm, irregular, non-tender swelling measuring approximately 4 x 5cm in diameter was palpable within the scrotal wall. It was distinct from the both testicles and spermatic cords, completely mobile and situated between the two testicles and pulling the scrotal skin downward due to the weight of the lesion. It appeared like a third testicle sitting between normal testicles. The overlying skin was normal and inguinal examination was unremarkable. Clinically labelled as sebaceous cyst and planned for surgical excision as patient was bothered by the heaviness due to increasing size. Both other testes were normal. Routine blood investigations were normal. Ultrasound revealed a 4 x 5 cm well-defined, hypoechoic extra testicular lesion arising from the scrotal wall, suggestive of a benign lesion. Complete surgical excision was performed under general anesthesia.

**Fig. 1:**
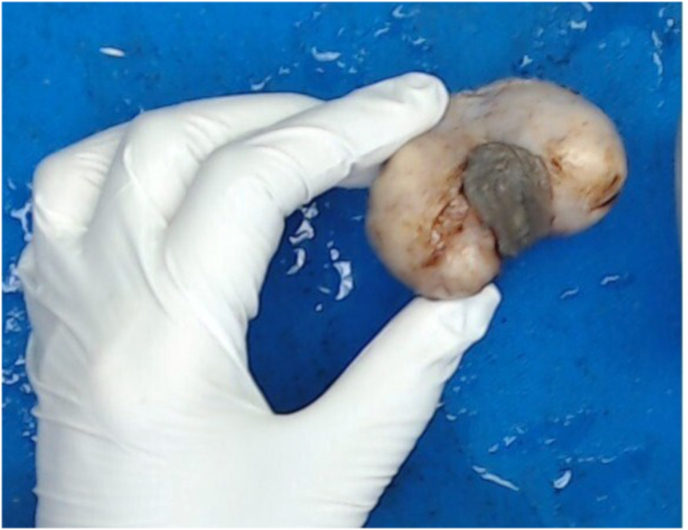
45mm × 35mm well circumscribed Lobulated pale and firm mass, completely excised from dartos (gross image from histopathology).

**Fig. 2:**
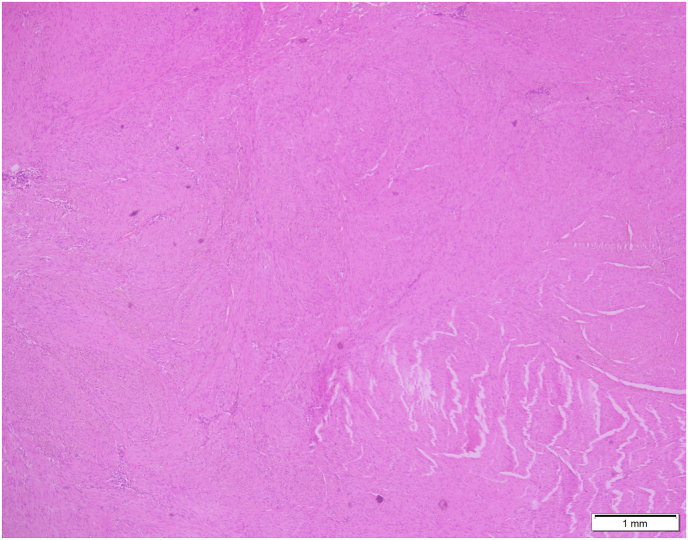
Spindle cells arranged in fascicles and bundles. (4x magnification).

**Fig. 3:**
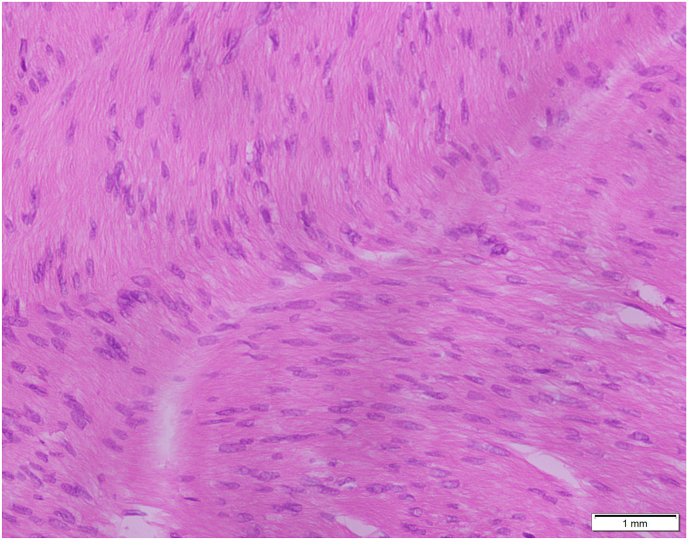
The Bland spindle cells are arranged in the form of whorls and interlacing fascicles, cigar shaped nuclei and abundant eosinophilic cytoplasm (40x magnification) (H&E Stain).

**Fig. 4:**
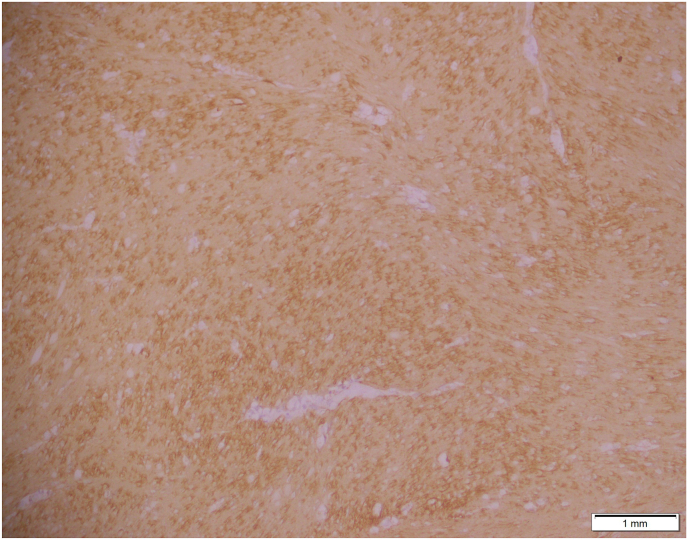
Smooth muscle actin showing cytoplasmic staining.

On surgical excision, a well-circumscribed, firm mass measuring 4-5 cm was identified within the dartos muscle (Fig. 1). Gross examination showed a firm, tan-white, whorled lesion. On serial slicing a single lobulated tan white hard cut surface lesion. Microscopy revealed interlacing bundles of spindle-shaped smooth muscle cells with elongated nuclei and eosinophilic cytoplasm. There was no atypia, necrosis, or mitotic activity, consistent with a benign leiomyoma (Fig. 2, Fig. 3). Immunohistochemistry staining with SMA was positive and SOX10 was negative, further confirming the smooth muscle origin and benign nature of the lesion respectively (Fig. 4).The postoperative course was uneventful. At 1 year follow-up, the patient remained asymptomatic with no recurrence.

## 3 Discussion

Leiomyomas were first described by Virchow in 1854 as benign soft tissue lesions arising from smooth muscles, first reported by Blanc in 1884.^5^^,^^6^ These can also develop in skin, scrotum, ovaries, bladder, renal pelvis, prostate, glans penis, lung, vascular structures, and spermatic cord.^7^ Fosters in 1858 first described scrotal smooth muscle tumors that arises from subcutaneous dartos muscle of the scrotum, and these account for less than 1% of scrotal tumors as reported by Siegel and Gaffy in a review of 11,000 cases.^8^ Primary scrotal leiomyomas are rare, with nearly 100 cases reported in the literature.^9^

They most commonly originate from the dartos muscle, though they may also arise from the cremaster muscle or vascular smooth muscle.^10^ Exact cause of these lesions remains unknown. Usually these present as chronic scrotal lesions present late, and longest duration reported is 30 years since onset,^11^ however our patient presented after 20 years. Noticing a scrotal mass is always concern for the patients, and they sometime assume it is likely to be malignant or infection related. At times men feel embarrassed to talk about scrotal abnormalities and report these late due to socio-cultural reasons. The good thing is that many painless scrotal masses are actually benign.

Clinically, they present as long-standing, painless, slow-growing scrotal swellings that mimic other benign lesions.^4^ On presentation scrotal leiomyoma can mimic adnexal tumors, adenomatoid tumors, neurofibroma, dermatofibroma and metastases. Schwannoma is considered a differential diagnosis if the mass is painful, and if there is skin ulceration, the likely cause is squamous cell carcinoma (SCC).^9^ In our case it appeared like a third testis, which is a very rare presentation. If it's a third testicle, then all testicles have same ultrasound echogenicity, whereas it was hypoechoic lesion arising from scrotal wall. Complete excision is both diagnostic and therapeutic. Recurrence is uncommon if excision is adequate.^12^

Imaging, particularly ultrasonography and MRI, can suggest a solid mass but lacks specificity in distinguishing leiomyomas from other neoplasms. Ultrasound is mostly the first line of imaging, and it reveals a well-circumscribed and heterogenous hypoechoic lesion. It is also clarifies extra testicular nature of lesion. MRI can be used in complex cases, and it shows an isointense on T1-weighted images, hypointense on T2-weighted images, and mild enhancement on post-contrast sequences.^13^^,^^14^

Histopathological confirmation is therefore essential. Microscopically typical leiomyomas demonstrate whorled bundles of spindle-shaped smooth muscle cells without nuclear atypia or mitotic activity [04, 13]. Leiomyomas have 2 main types i.e., typical and atypical based on histology. Leiomyomas are also graded as benign, atypical, or malignant depending upon 4 histologic features.^15^

i) Size ≥5 cm in greatest diameter
ii) Infiltrating margins
iii) ≥5 mitotic figures per 10 high power fields
iv) Moderate cytological atypia.

A tumor is classified as benign if it meets any one of the features, atypical if it meets any two, and leiomyosarcomas if it meets three or four of the above criteria. Benign and atypical lesions are treated by excision but atypical lesions require follow-up. Recurrence and malignant transformation is exceedingly rare in leiomyomas. However, leiomyosarcomas need aggressive management and long term follow-up.^16^ In our case, the tumor was 5 cm in its greatest dimension, with no atypia or mitotic figures without infiltrating margins. Hence, labelled as benign.

It is essential for a histopathologist to carefully assess for the any feature of malignancy, specifically the presence of mitotic activity, which signifies malignant potential. These lesions are most commonly found between the fourth decade and sixth decade of life in white men.^17^

Although renal cell carcinoma has been associated with cutaneous and uterine leiomyomas in autosomal dominant disorders such as HLRCC, but no association with scrotal leiomyomas has been established to date, which can be an avenue for molecular testing in future.^18^

Prior to excision, the patient was bothered by heaviness and dragging sensation, and expressed mild concern for sinister transformation, although the lesion had remained indolent for 20 years. He was relieved once the benign nature of the lesion was confirmed.

## 4 Conclusion

Long-term, painless scrotal swellings may be the first sign of primary scrotal leiomyoma. Both a definitive diagnosis and a cure can be achieved via surgical excision. Understanding this uncommon condition may help prevent incorrect diagnoses and ensure the best possible care.

## CRediT authorship contribution statement

**Vikram Lal Seetlani:** Data curation, Project administration, Writing – original draft. **Muhammad Waqas Shafiq:** Methodology, Resources, Writing – original draft. **Syed Adeel Ahmed:** Conceptualization, Supervision, Writing – review & editing. **Muhammad Bilal Rasheed:** Conceptualization, Data curation, Investigation, Methodology, Writing – original draft. **Faisal Rehman:** Methodology, Resources, Writing – review & editing. **Muhibullah Bangash:** Methodology, Supervision, Writing – review & editing. **Khurram Mir:** Conceptualization, Supervision, Writing – review & editing. **Mohammad Usman Khan:** Conceptualization, Writing – review & editing.
